# Optimization of peripheral blood volume for *in silico* reconstitution of the human B‐cell receptor repertoire

**DOI:** 10.1002/2211-5463.13467

**Published:** 2022-08-09

**Authors:** Hyunho Lee, Duck Kyun Yoo, Jerome Han, Ki Hyun Kim, Jinsung Noh, Yonghee Lee, Eunjae Lee, Sunghoon Kwon, Junho Chung

**Affiliations:** ^1^ Department of Electrical and Computer Engineering Seoul National University South Korea; ^2^ Department of Biochemistry and Molecular Biology Seoul National University College of Medicine South Korea; ^3^ Department of Biomedical Science Seoul National University College of Medicine South Korea; ^4^ Cancer Research Institute Seoul National University College of Medicine South Korea; ^5^ Interdisciplinary Program in Bioengineering Seoul National University South Korea; ^6^ BK21+ Creative Research Engineer Development for IT Seoul National University South Korea; ^7^ Bio‐MAX Institute Seoul National University South Korea

**Keywords:** antibody, B cell, B cell receptor repertoire, BCR‐based diagnostics, next‐generation sequencing, peripheral blood

## Abstract

B cells recognize antigens via membrane‐expressed B‐cell receptors (BCR) and antibodies. Similar human BCR sequences are frequently found at a significantly higher frequency than that theoretically calculated. Patients infected with SARS‐CoV2 and HIV or with autoimmune diseases share very similar BCRs. Therefore, *in silico* reconstitution of BCR repertoires and identification of stereotypical BCR sequences related to human pathology have diagnostic potential. Furthermore, monitoring changes of clinically significant BCR sequences and isotype conversion has prognostic potential. For BCR repertoire analysis, peripheral blood (PB) is the most convenient source. However, the optimal human PB volume for *in silico* reconstitution of the BCR repertoire has not been studied in detail. Here, we sampled 5, 10, and 20 mL PB from the left arm and 40 mL PB from the right arm of two volunteers, reconstituted *in silico* PB BCR repertoires, and compared their composition. In both volunteers, PB sampling over 20 mL resulted in slight increases in functional unique sequences (FUSs) or almost no increase in repertoire diversity. All FUSs with a frequency above 0.08% or 0.03% in the 40 mL PB BCR repertoire were detected even in the 5 mL PB BCR repertoire from each volunteer. FUSs with a higher frequency were more likely to be found in BCR repertoires from reduced PB volume, and those coexisting in two repertoires showed a statistically significant correlation in frequency irrespective of sampled anatomical site. The correlation was more significant in higher‐frequency FUSs. These observations support the potential of BCR repertoire analysis for diagnosis.

## Graphical abstract

Peripheral blood (PB) is the most convenient source for BCR repertoire analysis. However, the optimal volume for *in silico* analysis of the BCR repertoire has not been studied in detail. We sampled various volumes of human PB from different anatomical sites to compare the functional unique sequences that coexist among the repertoires in order to suggest optimal PB volume.

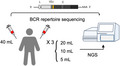

**Abbreviations**
BCR: B cell receptor
FUS: functional unique sequence
NGS: next generation sequencing
PB: peripheral blood
PBMC: peripheral blood mononuclear cell
SHM: somatic hypermutation
UMI: unique molecular identifier

B cells play a diverse role in the human immune system by recognizing a broad array of antigens via membrane‐expressed B‐cell receptors (BCR) and antibodies. The immense diversity of the BCR repertoire is generated by the rearrangement of V(D)J gene segments [1]. After antigen exposure, B cells undergo further diversification of the immunoglobulin (Ig) gene through somatic hypermutation (SHM) and class‐switch recombination in the germinal center (GC) [2, 3, 4]. The highly converted sequences diversify the structures, resulting in unique functions including specific antigen‐binding properties in variable regions or interactions with effector molecules by the constant region of the BCR [5]. Functionally determined BCR elicits immune responses, which could induce a protective or pathologic effect in multiple sites of the human immune systems [6]. By analyzing the BCR repertoire with next‐generation sequencing (NGS), it is conceivable to identify the panel of BCRs associated with the antigenic challenge, which could be used for diagnostic purposes and sometimes applied to the development of therapeutic antibodies [7].

Peripheral blood (PB) is the most common biological specimen to investigate the BCR repertoire for its accessibility compared to other sources that are difficult to acquire without invasive biopsy. In general, 1–50 mL of PB is sampled for further cellular enrichment and the NGS library preparation process [8, 9, 10, 11]. About 5 × 10^9^ B cells are circulating in approximately 5 L of PB (in a healthy adult human), which is about a 100–1000‐folds larger than the common sampling scale [12, 13]. To assess the experimental diversity of human PB BCR repertoire, Briney *et al*. [14] conducted the BCR sequencing at an extraordinary depth with a large number of circulating B cells harvested from the leukapheresis procedure. About 3 billion unique BCR sequences were generated from 10 healthy blood volunteers and about 10^8^ unique clonotypes were identified. In this study, the peripheral blood mononuclear cells (PBMC) were divided into six biological replicates and each biological replicate was used to prepare three technical PCR replicates. These biological and technical replicates showed quite similar V and J gene usage, suggesting that the BCR repertoire could be reconstituted *in silico* with little variation from PBMC. However, that study was limited in that it lacked the analysis on the correlation of BCR repertoire replicates at the level of full nucleotide sequences and isotypes determined by HCDR3 sequences and V/J genes. In another study, Simon *et al*. [15] collected 10 samples of 10 mL PB from two volunteers and generated the *IgG* repertoire. The results indicated that 40 mL of blood could guarantee reliable capture of the 1000 most common *IgGs* and the determination of their relative frequency in the circulation, while for the clonotypes with a lower abundance, increasing the PB volume could increase the overlap among the samples. However, the overlap did not increase monotonically, while the population of lower abundance clonotypes showed a greater deal of overlap than that of intermediate abundance clonotypes.

In this study we first tried to figure out the effect of PB volume as well as the depth of NGS on *in silico* BCR repertoire at the level of the functional unique sequences (FUS), defined as a unique full‐length BCR sequence and isotype at the nucleotide level. In detail, we investigated their effect on deriving FUS and Hill diversity of reconstituted the BCR repertoire. Thereafter, we studied how the relative frequency of FUS affects its presence and relative abundance in the BCR repertoire reconstituted from a lesser PB volume. In addition, we divided FUS into *IgM/D* along with other class‐switched isotypes and studied whether there is a difference.

## Materials and methods

### Ethics statement

The study was approved by the Institutional Ethics Review Board of Seoul National University Hospital (IRB approval number, 1801‐024‐913) and all blood samples were obtained after the volunteers had provided informed consent. All experiments were performed in accordance with the standards set by the Declaration of Helsinki.

### Blood sampling and RNA preparation

From two volunteers, PB was collected from both arms. From the left arm, 5, 10, and 20 mL of blood were drawn with an intermittent pause of 5 s. Right after the blood sampling from the left arm, 40 mL of blood was collected from the right arm. From the blood, peripheral blood mononuclear cells (PBMC) were isolated using Ficoll gradients (GE Healthcare, Chicago, IL, USA). Total RNA was isolated from PBMC using TRIzol reagent (15 596 018; Invitrogen, La Jolla, CA, USA) according to the manufacturer's protocol and used for the analysis.

### Next‐generation sequencing

Genes encoding the variable heavy chain (V_H_) and part of the heavy chain constant domain 1 (C_H1_) were amplified, using specific primers, as described previously [16]. Briefly, 1 μg of total RNA was used as a template to synthesize the first‐strand complementary DNA (cDNA), using the SuperScript IV First‐Strand Synthesis System (Invitrogen, Carlsbad, CA, USA), with reverse primers specific to the constant region (C_H1_ domain) of each isotype (*IgM*, *IgD*, *IgG*, *IgA*, and *IgE*) [16]. After the 1^st^ strand cDNA synthesis, 1.8 volumes of SPRI beads (AMPure XP, Beckman Coulter, Brea, CA, USA) were used to purify the cDNA, which was then eluted in 40 μL of water. The purified cDNA (18 μL) was subjected to the second‐strand synthesis in a 25‐μL reaction volume, using forward primer targeting V_H_ region [16] and DNA polymerase (KAPA HiFi HotStart, Roche, Basel, Switzerland). The reaction conditions were as follows: 95 °C for 3 min, 98 °C for 1 min, 55 °C for 1 min, and 72 °C for 5 min. Then, double‐stranded DNA (dsDNA) was purified using 1.0 volumes of SPRI beads. V_H_ genes were amplified with 15 μL of purified dsDNA, 2.5 pmol of the indexing primers (STM), and DNA polymerase (KAPA HiFi HotStart, Roche) in the thermal cycling program of 95 °C for 3 min; 25 cycles of 98 °C for 30 s, 65 °C for 30 s, and 72 °C for 1 min 10 s; and 72 °C for 5 min. PCR amplicons were gel purified using QIAquick gel extraction kit (Qiagen, Hilden, Germany) and then subjected to analysis by 4200 TapeStation System (Agilent Technologies, Santa Clara, CA, USA). The amplicons were pooled and subjected to NGS using MiSeq (Illumina, San Diego, CA, USA).

### Preprocessing and annotation of NGS data

The raw data from the NGS were preprocessed as described previously [16]. Briefly, paired‐end NGS reads were merged by PEAR with default parameters [17]. To filter out low sequencing quality reads, merged reads were selected if more than 95% of individual bases had a Phred score higher than 20. To remove sequencing error, a unique molecular identifier (UMI) within each read was identified, and read sequences were clustered based on the UMI sequence. The UMI cluster supported by more than two reads was selected, and the consensus sequence was constructed from each UMI cluster and used for further analysis.

The BCR sequence was aligned to the germline gene archived in the IMGT (the international ImMunoGeneTics information system) database using IgBLAST v1.8.0 [18, 19]. The number of V gene mutations was calculated by counting mismatches between the *IGHV* region sequence and aligned germline V gene sequence in the IMGT database. A sequence read successfully aligned with *IGHV*, and *IGHJ* gene and, at the same time, entailed the complete CDR1/2/3 sequence and isotype was called a functional read. Only the functional read was considered a reliable BCR sequence read and used for further analysis. We defined an FUS as a full‐length BCR nucleotide sequence with its own isotype. The analysis was performed at the individual FUS level. From each dataset of individual PB BCR repertoire, 190 000 reads were sampled and used for further analysis (Table S1).

### BCR repertoire analysis

Hill diversity of each PB BCR repertoire was analyzed using the inext r package version 2.1.7 [20]. For the overlap analysis, the BCR repertoire from 40 mL PB was considered as the reference, as it provided the highest diversity. The whole frequency range in the 40 mL PB BCR repertoire was divided into 10 equidistant sections on a logarithmic scale (Table S2). The coverage value of each section was obtained by dividing the number of FUS in 5, 10, or 20 mL PB BCR repertoire overlapping with 40 mL PB BCR repertoire by the number of FUS in 40 mL PB BCR repertoire. The representative frequency value of each section was calculated by the geometric mean of each section. In correlation analysis, overlapping FUSs were selected from two PB repertoires, and the correlation between frequency values within each repertoire were analyzed. Their association was determined by Pearson's correlation analysis using r (Vienna, Austria).

## Results

### Peripheral blood sampling strategy and reconstruction of BCR repertoire

From two healthy volunteers, we collected PB samples of 5, 10, and 20 mL from the left arm and 40 mL from the right arm (Fig. S1). From PBMCs, the total RNA was prepared and subjected to cDNA synthesis. The cDNA samples were used to amplify the gene fragments encoding V_H_ and a part of the C_H1_ region of the immunoglobulin heavy chain, which were analyzed in NGS. We could get over 2 million raw reads from all samples. The NGS data were preprocessed to annotate germline V/D/J gene usage, CDR 1/2/3 regions, isotype usage, and the number of V gene mutations. The number of functional reads after annotation reached over 190 000 in all samples (Table S1). For unbiased comparison, we sampled 190 000 functional reads from each sample. Although Hill diversity (*q* = 1) of the BCR repertoire was increased as the sampling volume of PB escalated, this increase became marginal after 20 and 10 mL in volunteers 1 and 2, respectively (Fig. S2). Hill diversity showed personal variation with a 2.5‐folder higher value in volunteer 2 compared to volunteer 1 in the 40 mL BCR repertoire.

**Fig. 1:**
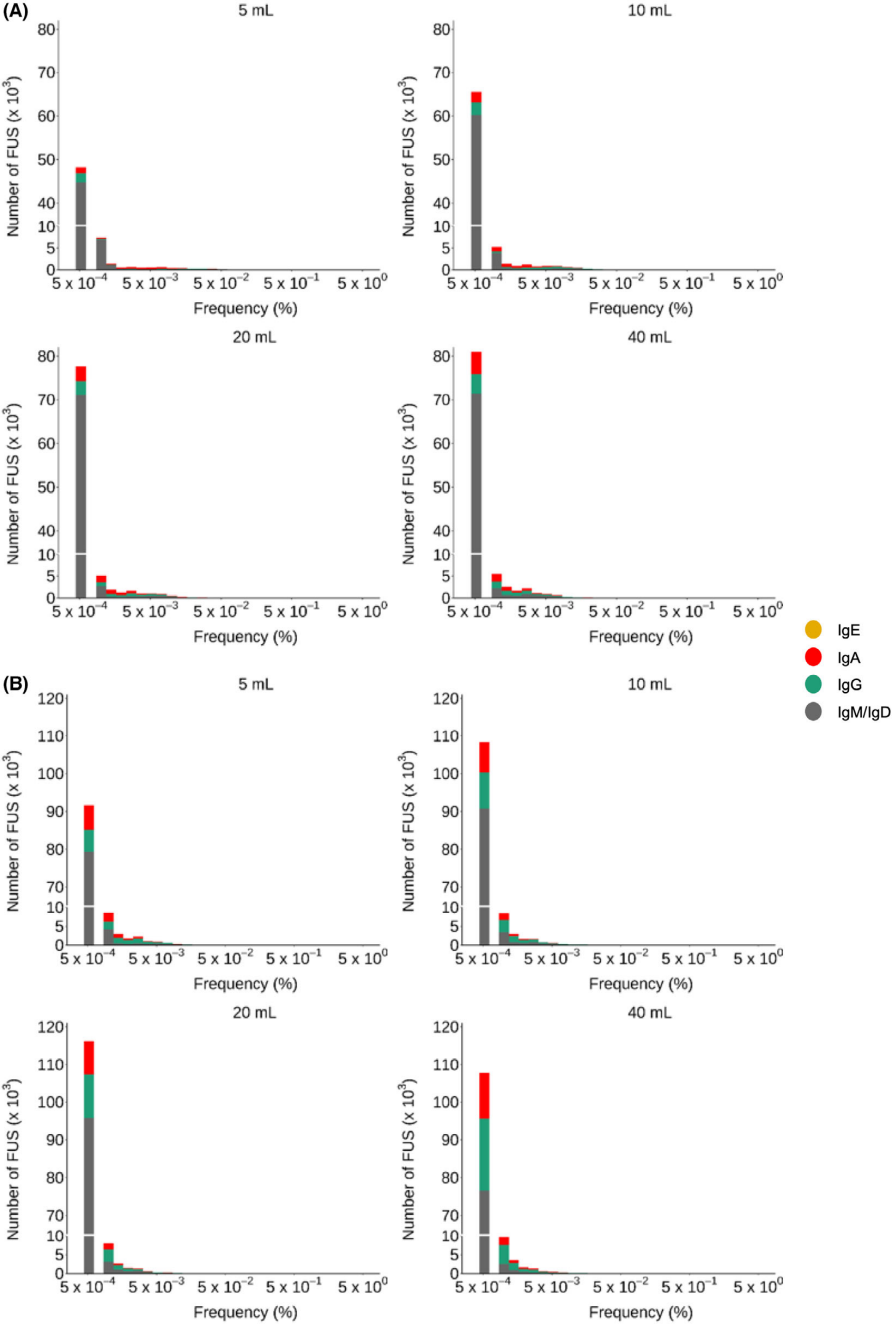
Frequency distribution of FUS in BCR repertoires constructed from PB. From two volunteers (A, volunteer 1; B, volunteer 2), 5, 10, 20, and 40 mL PB was sampled. The lowest frequency near 5 × 10^−4^ corresponds to one functional read. Using cDNA prepared from PBMC, V_H_ and a part of C_H1_ region gene were amplified for the NGS analysis. After annotation, FUSs were collected with their frequency. [Colour figure can be viewed at wileyonlinelibrary.com]

When FUSs were sorted with respect to frequency, it was clear that all BCR repertoires were dominated by FUSs with the lowest frequency value, which corresponded to one functional read, respectively (Fig. 1A,B). The major fraction of these FUSs was of the *IgM* isotype and probably originated from naïve B cells not exposed to antigenic stimuli. In healthy status, it was well known that PB B cells are dominated by naïve B cells with an *IgM* isotype [21], which gradually switches their isotype when facing antigenic challenge [16]. The increase of PB volume resulted in inflow of *IgM* or *IgD* FUSs with minor frequency value and an insignificant amount of somatic hypermutation (Fig. S3).

### Sampling PB volume‐dependent BCR repertoire coverage

**Fig. 2:**
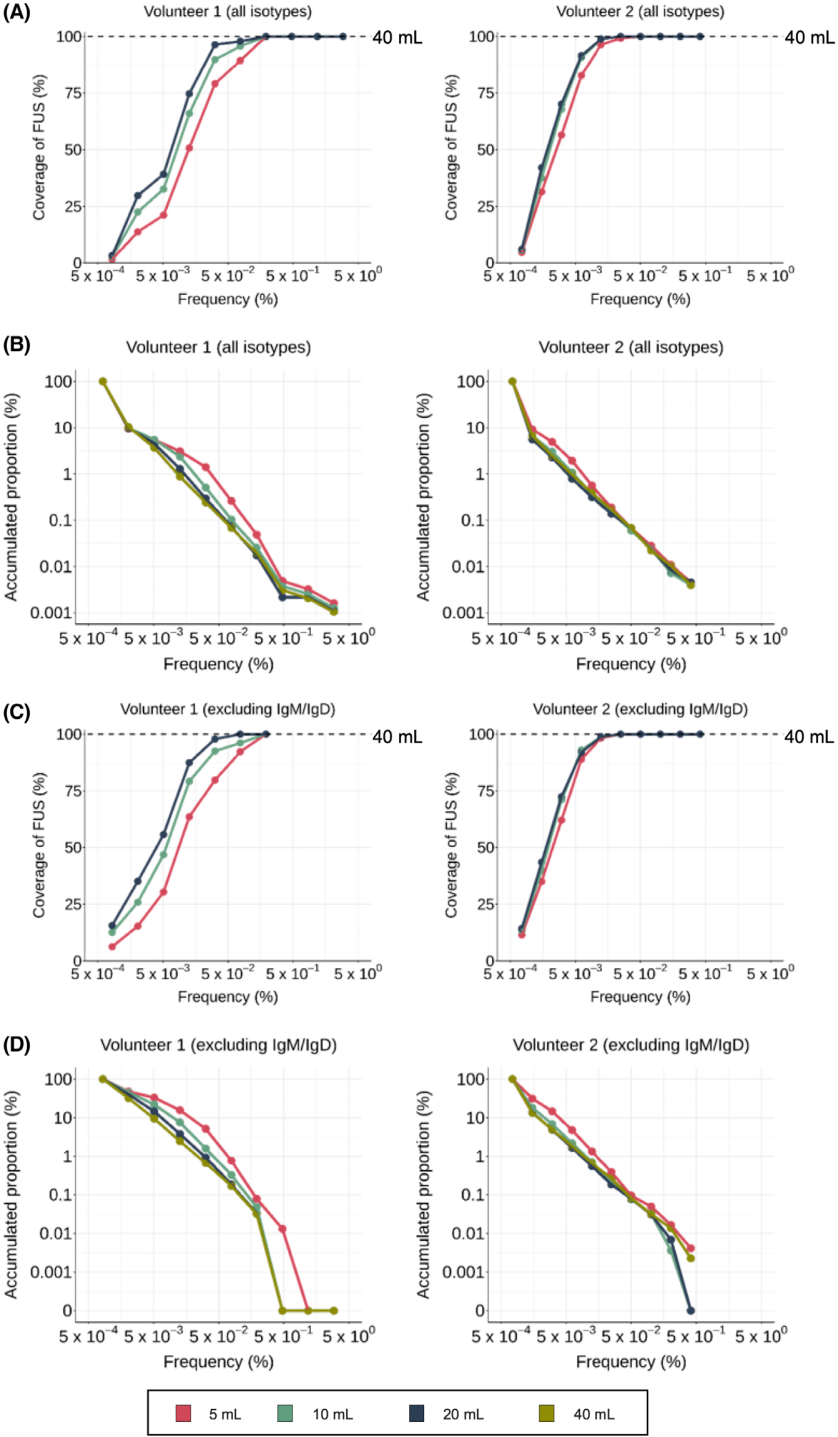
Coverage of FUS in 40 mL PB BCR repertoire at 5, 10, and 20 PB BCR repertoire. (A,C) FUS in the 40 mL repertoire were split into 10 sections based on their frequency. The coverage value of each section was obtained by dividing the number of FUS in 5, 10, or 20 mL PB BCR repertoire overlapping with the 40 mL PB BCR repertoire with the number of FUS in 40 mL PB BCR repertoire. The frequency value of each section was represented by the geometric mean of each section. Coverage measured using the entire isotypes (A) and after excluding *IgM/D* (C) were plotted. (B,D) The accumulated proportion of FUS in each section from the section with the highest frequency was plotted using the entire isotypes (B) and after excluding *IgM/D* (D). [Colour figure can be viewed at wileyonlinelibrary.com]

To check the distribution of overlapping FUSs among the PB BCR repertoire, FUSs in the 40 mL BCR repertoire were categorized into 10 sections based on their frequency on a logarithmic scale (Table S2). In each section, the number of FUSs found in 5, 10, and 20 mL BCR repertoires was identified. Then the coverage values of each section were obtained by dividing the number of FUS found in 5, 10, and 20 mL BCR repertoires by the number of FUS in that section of the 40 mL BCR repertoire. A 100% coverage was achieved in sections 7–10 in all 5, 10, and 20 mL BCR repertoires of volunteer 1 (Fig. 2A). The same coverage was achieved in sections 7–10, 6–10, and 6–10 in the 5, 10, and 20 mL BCR repertoires of volunteer 2, respectively (Fig. 2A). These sections contained 0.02% (section 7–10) and 0.39% or 0.17% (section 6–10 or 7–10) of FUS in the 40 mL BCR repertoire of volunteers 1 and 2, individually. In the rest of the sections, the coverage value decreased as the sampled PB volume decreased in both volunteers (Fig. 2A). In volunteer 1, above 50% coverage was maintained in sections 4–10 in all repertoires. The proportion of FUSs in section 4–10 corresponded to 3.1%, 2.4%, and 1.3% of the 5, 10, and 20 mL repertoire (Fig. 2B). In the case of volunteer 2, above 50% coverage was achieved in sections 3–10 in all repertoires. The proportion of FUSs in section 3–10 corresponded to 4.8%, 3.0%, and 2.2% of the 5, 10, and 20 mL repertoire.

Afterward, to focus on the BCR repertoire actively engaging in antigenic stimulus, we excluded *IgM* and *IgD* sequences from the BCR repertoires and analyzed the sequence coverage. A 100% coverage was achieved in sections 7–10 of all 5, 10, and 20 mL BCR repertoires of volunteer 1 (Fig. 2C), while it was achieved in sections 6–10 of all BCR repertoires for volunteer 2 (Fig. 2C). Above 50% coverage was maintained in sections 4–10, 4–10, and 3–10 of the 5, 10, 20 mL repertoire in volunteer 1 and in sections 3–10 of all repertoires in volunteer 2. The proportion of FUS accumulated corresponded to 33.1%, 7.6%, and 3.8% in the 5, 10, and 20 mL repertoire of volunteer 1 and 14.7%, 6.8%, and 4.9% in the 5, 10, and 20 mL repertoire of volunteer 2 (Fig. 2D).

### BCR sequence frequency is robust to sampling PB volume

**Fig. 3:**
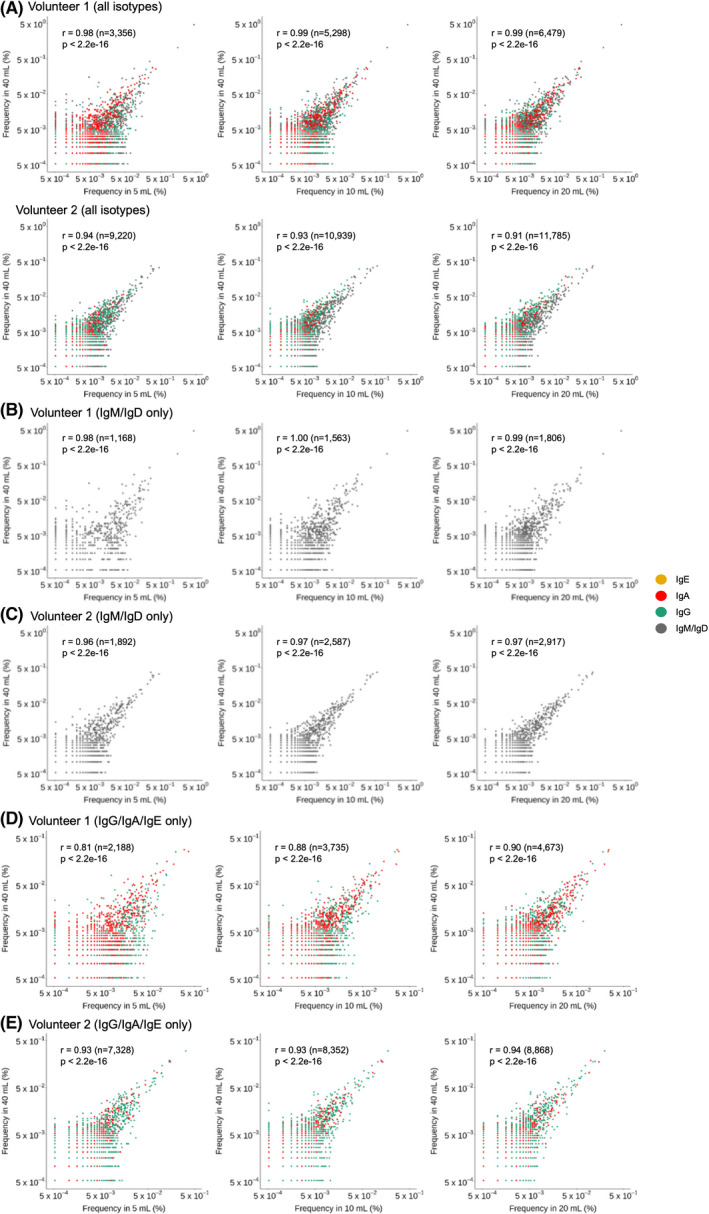
Correlation in the frequency of FUSs coexisting in two BCR repertoires. FUSs coexisting in 40 mL PB BCR repertoire and in repertoires prepared from a lesser volume of PB were plotted by their frequency in each repertoire. The plot was generated with all FUSs (A,B), FUSs with *IgM* or *IgD* isotypes (C,D), and FUSs with class‐switched isotypes (E,F). (A,C,E, volunteer 1; B,D,F, volunteer 2). [Colour figure can be viewed at wileyonlinelibrary.com]

To compare the BCR sequence frequency observed in different PB sampling volumes, we selected overlapping sequences and performed Pearson's correlation analysis for all pairs of all blood volumes in a pairwise manner. Comparisons between 40 mL with lesser volumes (Fig. 3) and those of the other pairs (Fig. S4) were analyzed. The correlation coefficients ranged from 0.96 to 0.98 in volunteer 1 (Figs. 3A and S4A) and from 0.86 to 0.93 (Figs. 3B and S4B) in volunteer 2 with a *P*‐value less than 2.2 × 10^−16^ in both volunteers, thus showing statistically significant correlations among BCR repertoires. It should be noted that the correlation coefficients of the 40 mL PB repertoire compared with the other PB repertoires were not significantly different from those compared among the 5, 10, and 20 mL PB repertoires. Also, the 40 mL PB samples were obtained from the right arm while the other PB samples were drawn from the left arm, confirming that PB B cells are distributed homogenously in the peripheral venous system. When we divided the BCR repertoires into the higher‐frequency repertoire and lower‐frequency repertoire with the cutoff frequency satisfying 50% coverage of the repertoire (Fig. 2A), the correlation coefficient among the higher‐frequency repertoire was 0.99 in volunteer 1 and ranged from 0.92 to 0.95 in volunteer 2 (Table S3). In the lower‐frequency repertoire, the correlation coefficient dramatically decreased with the range of 0.27–0.34 in volunteer 1 and 0.10–0.16 in volunteer 2. When only *IgM/D* repertoires are taken into consideration, the correlation coefficient ranged from 0.97 and 0.99 in volunteer 1 (Figs. 3C and S4C) and from 0.90 to 0.94 in volunteer 2 (Figs. 3D and S4D). On the other hand, when we exclude *IgM/D* sequences from the analysis, the correlation coefficients among PB repertoire decreased within the range between 0.62 and 0.83 in volunteer 1 (Figs. 3E and S4E), and between 0.82 and 0.89 in volunteer 2 (Figs. 3F and S4F).

The results of this study can be summarized in the following conclusions. First, increasing the PB sample volume could increase the number of FUS per functional read of NGS with a saturation pattern below 40 mL. Second, the Hill diversity of the PB BCR repertoire increased as the PB sample volume increased but similarly showed a saturation pattern below 40 mL. Third, as the sampling PB volume decreases, high‐frequency FUS has a higher chance to be found in the PB repertoire of lower PB volume, compared to FUS with relatively lower frequency. This pattern was more obvious in FUS with class‐switched isotypes. Fourth, the frequency of FUS found in PB repertoires from different PB volumes showed a statistically significant correlation. The correlation was more evident in FUSs with higher frequency.

## Discussion

BCR repertoires are shaped by continuous exposure to antigenic challenges. It was proved that its diversity is quite limited in germ‐free mice and can be expanded only after the exposure to a microbiome [22]. Through NGS analysis of PB BCR repertoire, we also showed that the antigen‐reactive BCR clonotypes are formed in chicken after immunization and booster [23]. Interestingly, it was found that humans share BCR sequences in a frequency much higher than that theoretically expected [24]. For example, we reported that a stereotypic V_H_ clonotype preexisted in a major fraction of the naïve human population, from which SARS‐CoV2 neutralizing antibodies could be raised in pairing with diverse light chains [16]. Other groups also reported stereotypic antibody clonotypes among COVID‐19 patients [25, 26, 27]. HIV‐1 was also reported to induce a very similar BCR sequence among patients [28] and the administration of immune checkpoint inhibitors induced very similar BCR sequences among the patients who responded to the treatment [29]. In addition, patients affected with autoimmune diseases such as rheumatoid arthritis and primary Sjögren's syndrome shares stereotypic rheumatoid factor [30, 31]. The presence of very similar antibodies among the population with a similar clinical situation inevitably proposes that BCR repertoire analysis can be performed for diagnostic purposes.

For diagnostic purposes, BCR sequences unique to the infectious or autoimmune diseases could be detected from the BCR repertoire constructed with an acceptable range of PB volume. Our study showed that BCR clonotypes with a frequency above 0.08% or 0.03% in 40 mL PB BCR repertoires could be detected in BCR repertoires from 20, 10, and 5 mL PB in volunteers 1 or 2, individually. The number of FUS above this threshold was 23 in volunteer 1 and 124 in volunteer 2. Many convergent BCR clonotypes among COVID‐19 patients showed a higher frequency than 0.03% [9]. The threshold is expected to be further lowered by performing NGS with a deeper depth as the number of unique FUS could be increased by obtaining more functional reads (Fig. S2). In this context, 5 mL PB might be sufficient in determine the infectious agents of patients. In our study, the number of FUS obtained from 20 mL PB was similar to that from 40 mL PB in both volunteers. Therefore, PB sampling over 20 mL would not be needed, which could be consolidated in the following studies with more volunteers.

Autoreactive antibody titers in autoimmune disease patients are known to fluctuate [32, 33] and their isotypes are altered as the disease progresses. For example, isotype changes to *IgG4* without the ability of complement activation and antibody‐dependent cell cytotoxicity could reduce disease progression [34, 35, 36]. Therefore, there are attempts to use it as a prognostic biomarker [37, 38, 39]. A limitation exists in such cases where autoreactive antibodies can be measured only by sophisticated diagnostic methods. For example, antibodies binding to acetylcholine receptor or muscle‐specific tyrosine kinase were found in ~80% and 1–10% of myasthenia gravis patients, respectively. The acetylcholine receptor is a membrane protein with multiple transmembrane domains and it is difficult to produce the recombinant acetylcholine receptor while persevering its native conformation [40, 41], which limits the accuracy of the related assays [42, 43]. Among the rest of the seronegative patients, autoantibodies to acetylcholine receptor were detected using a cell‐based assay requiring technical expertise and specialized equipment, which limits its general use [43]_._ In this context, monitoring the chronological changes in clinically significant BCR sequences like isotype conversion and frequency alteration can be an alternative and convenient option, although its value much depends on the accuracy of the analysis. In our study, FUS coexisting in two BCR PB repertoires showed a statistically significant correlation, irrespective of sampling anatomical site. The correlation was more significant in BCR sequences with higher frequency.

In summary, we report that the *in silico* BCR repertoire reconstitution, PB over 20 mL provides little more FUS in two volunteers. All FUSs with a frequency above 0.08% or 0.03% in the 40 mL PB BCR repertoire can be detected even with 5 mL PB. Additionally, the frequency of FUSs coexisting in two repertoires showed a statistically significant correlation as low as 5 mL PB, irrespective of sampling volumes or anatomical site, and the correlation was more significant in FUSs with higher frequency. Based on our study, the results suggest that 5 mL could be the least amount of PB volume for BCR repertoire analysis. All observations strongly propose the possibility to use BCR sequence information as a diagnostic measure.

## Conflict of interest

The authors declare no conflicts of interest.

## Author contributions

DKY designed and conducted all experiments, wrote and revised the article. HL analyzed sequencing data, visualized and interpreted results, and wrote and revised the article. JH interpreted analyses, wrote and revised the article. DKY, KHK, and EL participated in designing the experiment and recruited healthy volunteers. JN and YL analyzed sequencing data. SK conceived the study, designed and supervised the bioinformatics analysis. JC conceived the study, designed and supervised all experiments, interpreted all results, and wrote the article. All authors contributed to the article and approved the submitted version.

## Supporting information


**Table S1.** Statistics from NGS, annotation, and data processing.
**Table S2.** Section of FUSs in 40 mL PB BCR repertoire based on their frequency.
**Table S3.** Correlation of the frequency among FUSs coexisting in 40 mL PB BCR repertoire and those from lesser PB volume.
**Fig. S1.** Experimental schemes for blood sampling and NGS.
**Fig. S2.** Hill diversity of PB BCR repertoires.
**Fig. S3.** Somatic mutations on FUS.
**Fig. S4.** Correlation in the frequency of FUSs coexisting in two PB BCR repertoires.

## Data Availability

Raw BCR sequencing data are available under BioProject Accession PRJNA841058.
