# Supplementary material for: Optimization of peripheral blood volume for in silico reconstitution of the human B‐cell receptor repertoire
Source: FEBS Open Bio. 2022 Aug 9;12(9):1634–43. doi: 10.1002/2211-5463.13467 (PMC9433817; doi:10.1002/2211-5463.13467)
Supplement: Supplementary file 1 — Table S1. Statistics from NGS, annotation, and data processing. Table S2. Section of FUSs in 40 mL PB BCR repertoire based on their frequency. Table S3. Correlation of the frequency among FUSs coexisting in 40 mL PB BCR repertoire and those from lesser PB volume. Fig. S1. Experimental schemes for blood sampling and NGS. Fig. S2. Hill diversity of PB BCR repertoires. Fig. S3. Somatic mutations on FUS. Fig. S4. Correlation in the frequency of FUSs coexisting in two PB BCR repertoires. [file FEB4-12-1634-s001.docx]

***Supplementary Material***

**Analysis on the optimal peripheral blood volume for *in silico* reconstitution of human B cell receptor repertoire**

**Hyunho Lee^1†^, Duck Kyun Yoo^2,3†^, Jerome Han^2,3†^, Kihyun Kim^2^, Jinsung Noh^1^, Yonghee Lee^1^, Eunjae Lee^2,3^, Sunghoon Kwon^1,4,5,6^, Junho Chung^2,3,7^**

^1^Department of Electrical and Computer Engineering, Seoul National University, Seoul 08826, Korea.

^2^Department of Biochemistry and Molecular Biology, Seoul National University College of Medicine, Seoul 03080, Korea.

^3^Department of Biomedical Science, Seoul National University College of Medicine, Seoul 03080, Korea.

^4^Interdisciplinary Program in Bioengineering, Seoul National University, Seoul, 08826, Korea.

^5^BK21+ Creative Research Engineer Development for IT, Seoul National University, Seoul 08826, Korea.

^6^Bio-MAX Institute, Seoul National University, Seoul, 08826, Korea.

^7^Cancer Research Institute, Seoul National University College of Medicine, Seoul 03080, Korea.

^†^These authors contributed equally to this work.

*** Correspondence:**Sunghoon Kwon
[skwon@snu.ac.kr](mailto:skwon@snu.ac.kr)

Junho Chung
jjhchung@snu.ac.kr

**Supplementary Table 1.** Statistics from NGS, annotation, and data processing.

| Donor | Sampled Volume | Sampled  Site | Raw NGS reads | Functional read (sampled) | Number of FUS (sampled) |
| --- | --- | --- | --- | --- | --- |
| Volunteer 1 | 5ml | Left arm | 2,951,585 | 214,236 (190,000) | 67,568 (61,510) |
|  | 10ml | Left arm | 2,303,882 | 349,266 (190,000) | 130,430 (78,731) |
|  | 20ml | Left arm | 3,382,033 | 361,147 (190,000) | 158,384 (91,777) |
|  | 40ml | Right arm | 2,451,727 | 362,709 (190,000) | 166,984 (96,484) |
| Volunteer 2 | 5ml | Left arm | 2,750,952 | 386,942 (190,000) | 190,050 (110,272) |
|  | 10ml | Left arm | 2,554,736 | 313,147 (190,000) | 192,419 (125,158) |
|  | 20ml | Left arm | 2,743,957 | 198,764 (190,000) | 136,654 (131,349) |
|  | 40ml | Right arm | 2,197,348 | 343,679 (190,000) | 208,295 (125,813) |

**Supplementary Table 2.** Section of FUSs in 40 mL PB BCR repertoire based on their frequency

| Donor | Sections | Frequency Range (%) |
| --- | --- | --- |
| Volunteer 1 | 1 | 5.3×10^-4^ – 1.3×10^-3^ |
|  | 2 | 1.3×10^-3^ – 3.2×10^-3^ |
|  | 3 | 3.2×10^-3^ – 8.0×10^-3^ |
|  | 4 | 8.0×10^-3^ – 2.0×10^-2^ |
|  | 5 | 2.0×10^-2^ – 4.9×10^-2^ |
|  | 6 | 4.9×10^-2^ – 1.2×10^-1^ |
|  | 7 | 1.2×10^-1^ – 3.0×10^-1^ |
|  | 8 | 3.0×10^-1^ – 7.5×10^-1^ |
|  | 9 | 7.5×10^-1^ – 1.9×10^0^ |
|  | 10 | 1.9×10^0^ – 4.6×10^0^ |
| Volunteer 2 | 1 | 5.3×10^-4^ – 1.1×10^-3^ |
|  | 2 | 1.1×10^-3^ – 2.1×10^-3^ |
|  | 3 | 2.1×10^-3^ – 4.3×10^-3^ |
|  | 4 | 4.3×10^-3^ – 8.7×10^-3^ |
|  | 5 | 8.7×10^-3^ – 1.8×10^-2^ |
|  | 6 | 1.8×10^-2^ – 3.5×10^-2^ |
|  | 7 | 3.5×10^-2^ – 7.1×10^-2^ |
|  | 8 | 7.1×10^-2^ – 1.4×10^-1^ |
|  | 9 | 1.4×10^-1^ – 2.9×10^-1^ |
|  | 10 | 2.9×10^-1^ – 5.9×10^-1^ |

**Supplementary Table 3.** Correlation of the frequency among FUSs co-existing in 40 mL PB BCR repertoire and those from lesser PB volume.

| Volunteer | Section | Correlation coefficient | | |
| --- | --- | --- | --- | --- |
|  |  | 5 mL ~ 40 mL | 10 mL ~ 40 mL | 20 mL ~ 40 mL |
| Volunteer 1 | 1 – 7 | 0.99 | 0.99 | 0.99 |
|  | 8 – 10 | 0.22 | 0.35 | 0.41 |
| Volunteer 2 | 1 – 8 | 0.95 | 0.93 | 0.91 |
|  | 9 – 10 | 0.14 | 0.14 | 0.16 |


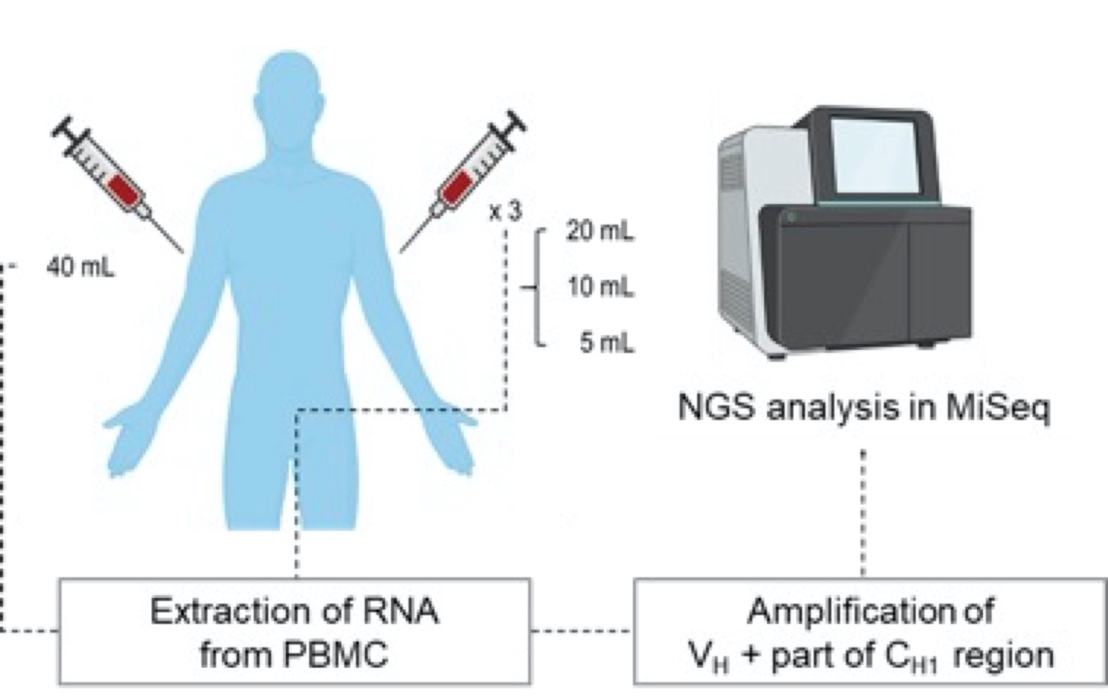


**Supplementary Figure 1.** Experimental schemes for blood sampling and NGS.

From two volunteers, peripheral blood was collected on both arms. From the left arm, 5 mL, 10 mL, and 20 mL of PB were drawn. Right after, 40 mL of PB was collected from the right arm. Using cDNA prepared from PBMC, V_H_ and a part of the C_H1_ region gene were amplified and subjected to NGS analysis.


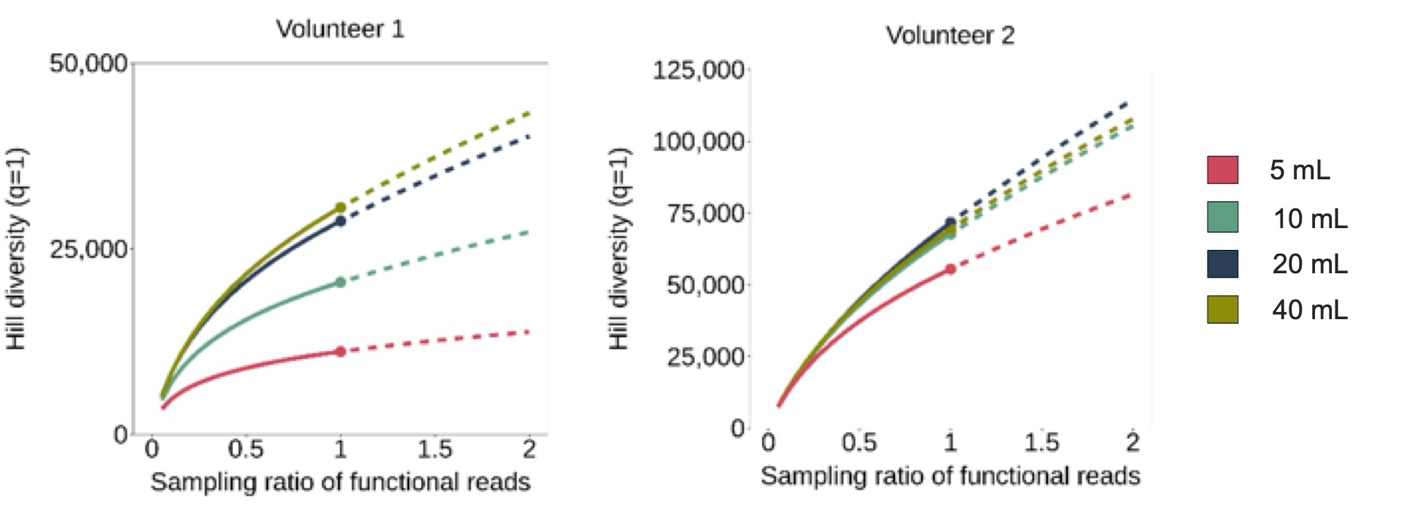


**Supplementary Figure 2.** Hill diversity of PB BCR repertoires.

Repertoire diversity was estimated using Hill numbers with the order of q=1. The solid line represented the rarefaction curve and the dashed line represented the extrapolation curve. Diversity estimate was calculated by increasing the fraction of repertoire subsampled. Diversity at sampling ratio equal to 1 was calculated by using the whole functional reads acquired by NGS. Potential diversity of the repertoire was estimated until the sampling ratio of 2 by extrapolation.


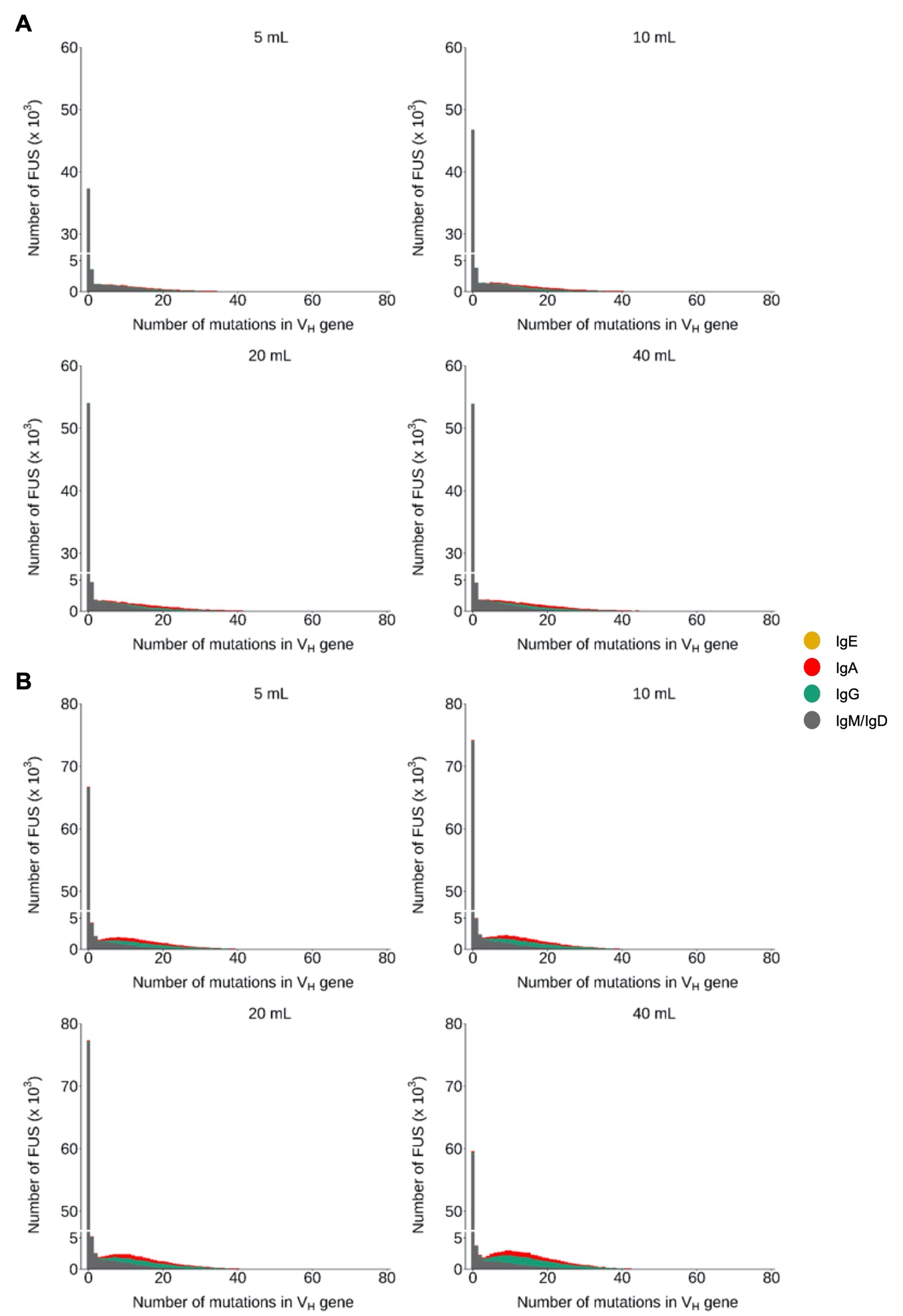


**Supplementary Figure 3.** Somatic mutations on FUS.

The number of somatic mutations in the IGVH gene of FUS was calculated. Then the number of FUS on each somatic mutation number was plotted. (A, volunteer 1; B, volunteer 2)


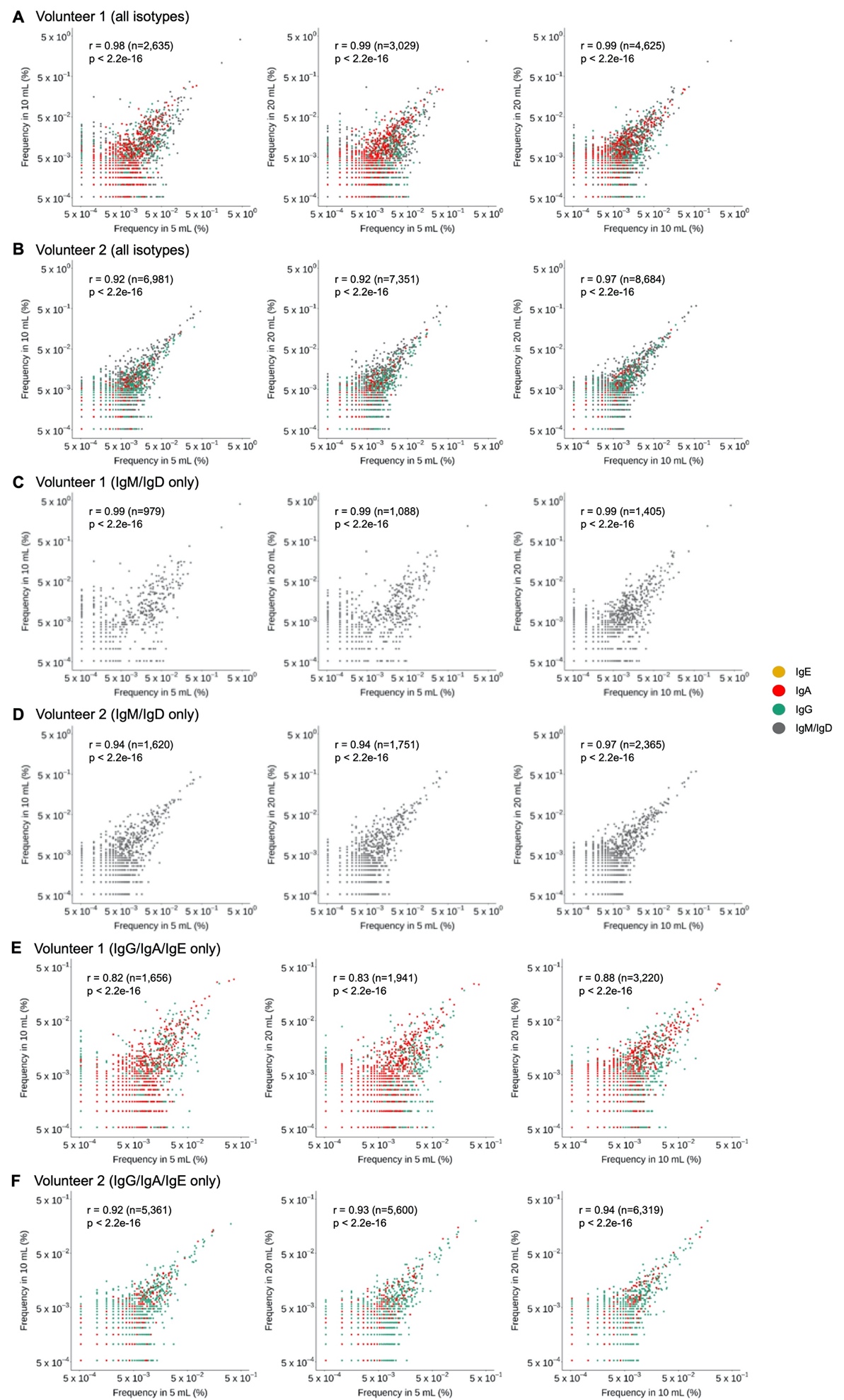


**Supplementary Figure 4.** Correlation in the frequency of FUSs co-existing in two PB BCR repertoires.

FUSs co-existing in two PB BCR repertoires were plotted by their frequency in each repertoire. The plot was generated with all FUSs (A & B), FUSs with IgM or IgD isotypes (C & D), and FUSs with class-switched isotypes (E & F). (A, C & E, volunteer 1; B, D & F, volunteer 2)
